# Metformin Inhibits the Growth of Hypopharyngeal Squamous Cell Carcinoma of Fadu Cell and Down-Regulates LncAROD to Improve Prognosis

**DOI:** 10.2174/0118715206427268251006112304

**Published:** 2025-10-29

**Authors:** Fang Liu, Huihui Xie, Meiyu Liu, Desheng Wang

**Affiliations:** 1 Department of Otolaryngology, Fujian Medical University Union Hospital, Fuzhou, 350001, China;; 2 Department of Otorhinolaryngology Head and Neck Surgery, First Affiliated Hospital of Gannan Medical University, Ganzhou, 341000, China;; 3 Department of Pathology, First Affiliated Hospital of Gannan Medical University, Ganzhou, 341000, China;; 4 The First Clinical Medical College, Gannan Medical University, Ganzhou, 341000, China

**Keywords:** Metformin, hypopharyngeal squamous cell carcinoma (HSCC), fadu cell line, lncAROD, cancer biomarkers, prognosis

## Abstract

**Background:**

Hypopharyngeal Squamous Cell Carcinoma (HSCC) is associated with a poor prognosis due to challenges in early detection, early metastasis, and limited treatment options.

**Objective:**

This study aims to investigate the effect of metformin on HSCC and identify potential prognostic factors associated with this carcinoma.

**Methods:**

The effects of metformin in HSCC cells were tested by functional assays *in vitro*. A xenograft tumor model was established, which was further examined by H&E staining, immunohistochemistry, and transmission electron microscopy (TEM). RNA sequencing analysis was employed to investigate the effects of metformin on gene expression and associated pathways. Bioinformatic analysis was further conducted to elucidate potential mechanisms and their correlation with gene expression, the tumor immune microenvironment, and survival prognosis. Finally, we further assessed the effect on FaDu cells by knocking down lncAROD using siRNAs.

**Results:**

The results demonstrated that metformin significantly reduced cell viability and migration, while promoting apoptosis and inducing cell cycle arrest in FaDu cells. WB analysis revealed that metformin inhibits the development of FaDu cells, possibly through the EMT pathway. *In vivo* studies indicate that metformin effectively inhibits tumor growth, promotes apoptosis, and autophagy. RNA-seq analysis revealed that metformin led to the upregulation of 1,697 genes and the downregulation of 858 genes, particularly highlighting a significant reduction in lncAROD, which were subsequently verified by qRT-PCR. Bioinformatic analysis demonstrated that lncAROD is highly expressed, with patients exhibiting higher levels of lncAROD showing poorer prognoses. Knockdown of lncAROD can reduce the proliferation, migration, and invasion of FaDu cells.

**Conclusion:**

This finding presents a novel approach to the clinical management of HSCC, indicating that metformin influences various processes related to the growth and progression of HSCC. Specifically, it reduces lncAROD expression and inhibits tumor progression, suggesting that lncAROD may serve as a valuable biomarker for evaluating the prognosis of HSCC.

## 1 INTRODUCTION

Head and neck cancer ranks as the seventh most frequently diagnosed cancer globally [1], with hypopharyngeal squamous cell carcinoma (HSCC) exhibiting the worst prognosis among these cases [2], characterized by a 5-year overall survival (OS) rate of merely 30-40% [3]. Nevertheless, the treatment of this condition continues to face numerous challenges and obstacles [4]. Early diagnosis of HSCC is essential due to its gradual onset, and the vague symptoms of this condition frequently result in diagnosis at advanced stages, complicating the treatment process [5]. Currently, clinical practice is hindered by a distinct lack of effective early detection approaches, resulting in numerous patients missing their best opportunity for treatment [6]. Conventional HSCC therapies, including operative procedures, radiation exposure, and chemical agents, often result in substantial complications that detrimentally influence patients' quality [7]. Additionally, conventional therapies may not only fail to produce satisfactory outcomes in patients with advanced HSCC but also increase the likelihood of recurrence and metastasis [8]. Consequently, it is essential to clarify the underlying mechanisms of HSCC further and develop more efficient treatment options [9, 10].

Drug repurposing offers distinct advantages in cancer treatment, as it is typically cost-effective, has demonstrated a safe profile, and can significantly accelerate the drug development process due to its established safety framework [11, 12]. Over 200 non-anticancer drugs have demonstrated antitumour effects as off-label indications [13]. These drugs act through diverse mechanisms, including metabolic modulation, immune regulation, angiogenesis inhibition, and induction of apoptosis [14, 15]. For instance, the antidepressant sertraline has been shown to inhibit the growth of breast cancer cells by inducing apoptosis and cell cycle arrest [16]. Advances in multi-omics technologies and AI-based predictive models have facilitated the systematic identification of repurposed non-oncologic drugs with anti-tumor potential, thereby further accelerating the development of precision oncology [17, 18].

Solid Tumors Microenvironment (TME) exhibits significant heterogeneity, leading tumor cells to enhance their glucose absorption for energy production [19]. Metformin exerts its antihyperglycemic action by downregulating key enzymatic processes involved in liver glucose output, specifically gluconeogenesis and glycogen breakdown [20]. Metformin is also considered a potential therapeutic agent for cancer treatment [21]. Studies suggest that this medication may inhibit tumor cell growth and proliferation, trigger apoptosis, and prevent both metastasis and invasion [22-24]. The observed effects appear to be mediated through the AMP-activated protein kinase (AMPK) pathway, a central modulator of cellular energy homeostasis [25]. Metformin successfully hinders tumor growth by altering the energy equilibrium of tumor cells and reducing their glucose absorption and metabolic processes [26, 27]. Multiple studies have suggested that metformin has the potential to improve the effectiveness of chemotherapy in cancer cells, enhance responsiveness to chemotherapeutic agents, and amplify the impact of immunotherapeutic treatments [28-30].

Cancer is characterized by metabolic alterations, with the reconfiguration of energy metabolism being a well-acknowledged occurrence in tumor biology [31-33]. Research indicates that lncRNAs significantly influence the regulation of energy metabolism in cancer patients [34]. The increased levels of particular lncRNAs in tumors have been linked to the seriousness of the disease and its prognosis [35, 36]. For instance, MALAT1 and HOTAIR exhibit high expression levels in multiple tumors, correlating with tumor growth, invasion, and spread [37, 38]. Additionally, H19 enhances the autophagy response, exhibits a positive correlation with several drug treatments, and may serve as an effective therapeutic target for various types of cancers, thereby substantially improving patient prognosis and clinical outcomes [39]. As a result, the potential to effectively impede tumor progression through the modulation of these lncRNAs suggests the development of novel cancer treatment strategies.

Transcriptome sequencing serves as a powerful tool for comprehensively profiling gene expression in tumor cells, enabling the detection of genomic alterations and key genes implicated in tumor pathogenesis [40]. Bioinformatics-based prognostic analysis has become indispensable in clinical oncology for developing personalized treatment strategies [41]. By integrating multidimensional data-including genotypic, phenotypic, and clinical information—this approach significantly improves the assessment of tumor aggressiveness, metastatic potential, and prognostic outcomes, thereby establishing a robust scientific foundation for precision oncology [42]. In a study, Zheng *et al*. employed transcriptome sequencing to uncover a network of interactions between lncRNA and mRNA linked to the disease [43]. Tian *et al*. conducted a bioinformatics analysis to investigate the role of aldolase A in tumor prognosis [44]. We screened the results of transcriptome sequencing and performed bioinformatics analyses to elucidate the relationships between the expression of different genes and clinicopathological features, as well as prognosis.

This study demonstrated that metformin exerts an anti-tumor effect on FaDu cells, and its efficacy is correlated with the cellular level of lncAROD. Additionally, we assessed in vivo tumorigenicity through subcutaneous xenografts to examine drug toxicity and its impact on tumor development.

## 2 MATERIALS AND METHODS

### 2.1 Cell Culture and Processing

The FaDu cell line is a hypopharyngeal squamous cell carcinoma that used most widely, and it was obtained from Haixing Biology (Suzhou, China). These cells were cultured in complete medium containing 10% FBS and 1% penicillin. The cell culture environment was maintained at 37°C with 5% CO_2_ concentration and adequate humidity.

### 2.2 Reagents and Drugs

The metformin used in this study was purchased from North China Pharmaceutical Co., Ltd. For experimental use, a 700 mM stock solution was made by dissolving 100 mg of metformin in RPMI 1640 medium.

### 2.3 Cell Viability (CCK-8)

To evaluate cell viability, a CCK-8 assay was conducted. FaDu cells were plated at 2×10^4^ cells/well(100 μL medium) in 96-well plates. After metformin exposure (graded concentrations; 24-72 h), 10 μL of CCK-8 solution was added per well. Following a 60-minute incubation, spectrophotometric measurements at 450 nm enabled the calculation of viability relative to untreated controls.

### 2.4 Colony Formation Assay

Cells were grown in 6-well plates at a density of 1000 cells per well, while being subjected to different concentrations of metformin (0, 10, 20, and 40 mM). In a subsequent experiment investigating the combined effects of siRNA-lncAROD and metformin on cell proliferation, the cells were divided into four groups:NC, NC+metformin, siRNA-lncAROD, and siRNA-lncAROD+ metformin. Throughout a duration of 2 weeks, the complete culture medium changes were performed every 72 hours alongside regular cell monitoring. Colonies were then fixed in methanol and crystal violet-stained (Beyotime, China). After capturing the images using a microscope (Olympus, Tokyo, Japan), the colony count was carried out using ImageJ.

### 2.5 Wound Healing Assay

FaDu cells (6 × 10^5^) were seeded into 6-well plates and cultured for 24 hours. Following this initial incubation, both control and metformin-treated groups were exposed to either 0 or 10 mM of metformin for an additional 24 hours. A sterile 10-μl pipette tip was then used to create a scratch wound in the cell monolayer, and the location was marked for imaging. Subsequently, the cells were washed three times with PBS to remove non-adherent cells and debris, followed by continued incubation at 37°C under 5% CO_2_. At specified time intervals (24, 48 and 72 hour), the cells were collected, examined microscopically, and photographed. The extent of cell movement was assessed by measuring the width of the wound. A red line was drawn following the image capture to facilitate the measurement of the distance between the scratched cells.

### 2.6 Flow Cytometric Analysis

Cell apoptosis was assessed using the TransDetect^®^ Annexin V-FITC/PI Apoptosis Detection Kit (TransGen, China). Following a 48-hour treatment with metformin, FaDu cells were harvested and rinsed with cold PBS. The cells were then resuspended in 1× Annexin V Binding Buffer and stained with Annexin V-FITC and propidium iodide (PI). The staining procedure was conducted at room temperature in the dark for 15 minutes, as per the manufacturer’s instructions. Apoptotic cells were quantified using a BD FACSCanto II flow cytometer, with further analysis of the data analysis conducted using FlowJo 7.6.

FaDu cells treated with varying concentrations for 48 hours were analyzed for cell cycle distribution using the red fluorescent Elabscience^®^ Cell Cycle Assay Kit. After the treatment, the cells underwent digestion, centrifugation, washing, and were subsequently fixed in anhydrous ethanol at -20°C for one hour. Once fixation was complete, the cells were incubated with a PI solution in the dark at 4°C for 30 minutes prior to analysis using a flow cytometer (BD, USA).

### 2.7 Western Blotting

FaDu cells treated with metformin were lysed using RIPA lysis buffer, after which the protein concentration was assessed through a BCA protein assay. Twenty micrograms of protein were separated using SDS-PAGE and subsequently transferred to a PVDF membrane. The membranes were blocked with 5% nonfat milk and incubated with primary antibodies targeting E-cadherin (1:1000, Proteintech, China), N-cadherin (1:1000, Proteintech, China), and GAPDH (1:1000, Proteintech, China). Following this, the blots were detected using HRP-coupled secondary antibodies (1:5000, Proteintech, China), and the resulting chemiluminescence signals were quantified using ImageJ. All experiments were executed with three independent replicates (Supplementary Material).

### 2.8 Xenograft Models

Ten 5-week-old female BALB/c nude mice were acquired from Jiangsu Huachuang Sino Pharma Tech Co., Ltd. and housed in specific pathogen-free (SPF) conditions at Gannan Medical University's Animal Experiment Center. Mice were housed at 25±2°C with 40-60% humidity under a 12-h light/dark cycle (lights on at 08:00), with free access to a standard rodent diet and water. For tumor xenograft establishment, 5×10^6^ FaDu cells in suspension were administered *via* subcutaneous injection in the flank area. After about 14 days, treatment was initiated when the tumor volume reached approximately 180 mm³. Tumor size was assessed manually by measuring two perpendicular dimensions using a digital caliper. The approximate tumor volume was calculated using the widely accepted formula: Tumor Volume (mm³) = (Length × Width²) / 2.The animals were randomly allocated into two treatment groups(n=5 per group): one receiving daily metformin (20 mg/kg, i.p.) and the other receiving equal volumes of saline (i.p.). The sample size was minimized while ensuring statistical robustness, in compliance with ARRIVE guidelines, and was approved by the Animal Ethics Committee of Gannan Medical University.

To alleviate potential pain, mice were maintained under gaseous anesthesia (isoflurane in oxygen) throughout all experimental manipulations. After a continuous treatment and evaluation period of 30 days, the mice were euthanized through CO_2_ inhalation. Tumors were surgically removed for volumetric and gravimetric assessment, while tumor and liver specimens were preserved for additional analyses.

All experimental procedures involving animal care, handling, and euthanasia strictly adhered to ARRIVE guidelines and were performed in compliance with institutional animal care policies as well as national and international regulations on laboratory animal welfare. The Animal Ethics Committee of Gannan Medical University approved all animal experiments.

### 2.9 H&E Staining and Immunostaining Analysis

Tumor specimens were bisected, with one portion allocated for total RNA isolation and the remainder processed for histological evaluation, which included both H&E staining and Ki-67 immunohistochemical examination. Hepatic tissues collected from nude mice were histologically analyzed through hematoxylin and eosin (H&E) staining. Tissue specimens were subjected to a 48-hour treatment with 4% paraformaldehyde, after which they were embedded in paraffin and subsequently stained using H&E. The IHC staining was performed using the automated Ventana BenchMark XT (Roche Diagnostics, Basel, Switzerland) with a Ki-67 dilution of 1:200 (Roche).

### 2.10 Transmission Electron Microscopy(TEM)

Neoplastic specimens were collected and immediately fixed in TEM-grade preservative (Servicebio, China) at 4°C to maintain ultrastructural integrity during storage and transportation. Processed tissue samples were subsequently examined under a Hitachi HT7700 transmission electron microscope (Tokyo, Japan) following standard protocols, with images observed for autophagosome and mitochondrial structure.

### 2.11 RNA Sequencing and Data Analysis

FaDu cells (1×10^6^) were exposed to 10 mM metformin for 48 hours *in vitro*, using three independent replicates. Both the experimental and control groups consisted of three independently treated samples. Following this treatment, the cells were collected for subsequent RNA sequencing analysis. To extract total RNA from the samples, we utilized the TRIzol reagent from TransGen Biotech, ensuring the reliability of our results through the evaluation of RNA integrity using the Agilent 2100 RNA Nano 6000 Assay Kit. The preparation of high-throughput RNA sequencing libraries was constructed using the AMPure XP system, and the quality of these libraries was assessed with an Agilent 2100 bioanalyzer. The Illumina NovaSeq 6000 system (Annoroad Medical Testing, Beijing) was employed for sequencing analysis. To analyze the data resulting from the sequencing, we quantified gene expression and identified differentially expressed genes using the FeatureCounts and DESeq2 R packages. In doing so, we adhered to established threshold criteria, specifically a false discovery rate (adjusted *p*-value) of ≤ 0.05 and a log2 fold change of ≥ 1.

We then performed Gene Ontology (GO) and Kyoto Encyclopedia of Genes and Genomes (KEGG) enrichment analyses according to the protocols outlined in the clusterProfiler R package. Additionally, gene set enrichment analysis (GSEA) was also conducted.

### 2.12 qRT-PCR Analysis

To investigate metformin's influence on lncAROD expression, FaDu cells cultured in RPMI-1640 medium supplemented with 10% FBS were divided into two groups: treated with metformin (10 mM) or PBS control for 24h. RNA from cells and xenografts was extracted using TRIzol (TransGen) and analyzed by qRT-PCR (PerfectStart kit, TransGen), with GAPDH-normalized 2^-ΔΔCt^ quantification.

Gene-specific primers were produced by Sangon Biotech (China), the sequences of the primers are provided:lncAROD, 5’-CCACAACGGCAACCAGTAAA-3’(Forward primer) and 5’-AGGCGTTCCACCTGCAAATA-3’(Reverse primer) and GAPDH, 5’-CAGGAGGCATT GCTG ATGAT-3’(Forward primer) and 5’-GAAGGCTGGGGCTCATTT-3’ (Reverse primer). Supplement Table **S2** contains the primers for other genes.

### 2.13 Bioinformatics Analysis

Due to the absence of a specific database for HSCC, a typical subtype of head and neck squamous cell carcinoma (HNSCC), we utilized data available from the HNSCC database. The RNA sequencing data alongside clinical details for HNSCC patients were sourced from the TCGA database. This study incorporated a total of 302 HNSCC samples along with 44 paracancerous tissue samples. The RNA sequencing datasets, originally quantified in FPKM units, were converted to TPM (transcripts per million) values and log2-transformed for downstream analysis. Furthermore, we obtained additional TPM-formatted RNA-seq data from the UCSC Xena database, encompassing both TCGA and GTEx datasets, both of which underwent uniform processing through the Toil process. This publicly accessible database is exempt from Institutional Review Board approval requirements.

For the analysis of the correlation between the lncAROD-associated gene and other genes, we utilized the stats package available in R. The top 200 genes showing the strongest positive Pearson correlations with lncAROD were identified for subsequent functional characterization. Both GO enrichment and KEGG pathway analyses were performed on these top 200 positively associated genes using the "ClusterProfiler" R package, with visualizations created through the "ggplot2" package. P-values below 0.05 were deemed statistically significant during the univariate Cox analysis, which was performed using the R package 'survival.'

A comparative analysis was conducted between HNSCC tissues and matched adjacent normal tissues using both parametric (paired t-test) and non-parametric (Mann-Whitney U test) statistical approaches. ROC curve analysis was performed using the pROC package to establish optimal lncAROD cutoff values. Associations between lncAROD expression and clinicopathological parameters were assessed through Wilcoxon rank-sum tests, Fisher's exact tests, chi-square tests, and logistic regression models. The influence of lncAROD on survival outcomes was examined using Kaplan-Meier plots alongside log-rank tests. For both univariate and multivariate analyses, Cox proportional hazard models were utilized to explore the connections between clinical features and OS.

### 2.14 siRNA-lncAROD Transfection

siRNA aimed at lncAROD (referred to as siRNA-lncAROD), along with a scrambled siRNA intended as a negative control, were procured from Shanghai GenePharma, China. FaDu cells underwent transfection with the specific siRNA using siRNA-mate plus(also from Shanghai GenePharma, China), adhering to the guidelines provided by the manufacturer. The siRNA concentration delivered into the cells was 10 ng/µl in a 6-well plate setup. Cells were seeded at a density of 60% 24 hours prior to transfection, after which subsequent experiments were conducted. The siRNA sequences are provided in Table **S3**.

### 2.15 Migration and Invasion Assays

The assessment of the migration and invasion abilities of FaDu cells transfected with siRNA-lncAROD was conducted using a transwell assay. FaDu cells were placed in serum-free media located in the upper chamber of a 24-well Transwell Chamber (8 μm pore size, Corning, USA), either uncoated or coated with Matrigel (Servicebio, China). The chambers were incubated for 48 hours in a culture medium containing 10% FBS in the lower chamber. Non-migrated and non-invaded cells were subsequently removed using cotton swabs. Finally, the invading cells were fixed (4% PFA), stained (0.1% crystal violet, 10 min), and quantified microscopically. Cell counts were determined using ImageJ software for objective analysis.

### 2.16 Statistical Analysis

All experiments were performed with a minimum of three replicates, and data are presented as mean±standard deviation (SD). Statistical analyses were conducted using GraphPad Prism 8.0, SPSS 20.0, and R version 3.6.3 Intergroup differences were assessed by one-way analysis of variance or unpaired t-tests, with a p-value less than 0.05 considered statistically significant. The graphical abstract was created with Figdraw.

## 3 RESULTS

### 3.1 Metformin Reduces Viability, Migration and Proliferation of FaDu Cells

To assess metformin's impact on FaDu cell biology, we conducted comprehensive analyses of cellular viability, proliferative capacity, and migratory potential. Cell viability was measured using the CCK-8 assay after treatment with gradient concentrations of metformin over 24-72 hours. Clonogenic survival and scratch wound assays were employed to evaluate proliferation and migration, respectively. Results indicated that metformin reduced the proliferation of FaDu cells in a dose-dependent manner, with a calculated IC50 value of 10 mM at the 48-hour mark (Fig. **1A**). Based on preliminary results, metformin concentrations ranging from 0 to 40 mM were chosen for further functional analyses. Wound healing assays demonstrated markedly reduced migratory capacity of FaDu cells at 48 and 72 hours post-metformin treatment, as quantified in Fig. (**1C**) (representative images shown in Fig. **1B**). The plate colony formation assays demonstrated that metformin treatment led to fewer colonies (Fig. **1D**). Additionally, higher metformin concentrations resulted in a significant decline in cell aggregation and clone formation (Fig. **1E**). The data clearly show metformin's antiproliferative effects on FaDu cells. Dose-response experiments additionally confirmed its ability to attenuate HSCC cell biological functions in a concentration-dependent fashion.

### 3.2 Metformin Initiates Apoptosis and Arrests Cell Cycle Progression

To investigate the effects of metformin on FaDu cells, flow cytometric evaluations of apoptosis were conducted after the cells were exposed to metformin solutions of 0mM, 10 mM, 20 mM, and 40 mM for 48 h. The results showed a significant increase in the apoptosis rate of FaDu cells following metformin treatment, *p* < 0.001 (Fig. **2A**). Higher metformin concentrations resulted in a progressive increase in the apoptosis rate in Fadu cells, with a statistically significant rise in the rate of apoptotic cells from 8% to 44% (*p*< 0.05). These findings suggest that metformin promotes apoptosis in FaDu cells and that the apoptosis level is positively associated with metformin concentration (Fig. **2C**). Additionally, a substantial rise was observed in apoptosis at metformin concentrations of more than 20 mM, as the majority of cells were dead at the 40 mM treatment.

This study also examined the effects of metformin on cell cycle progression to further clarify the relationship between cell cycle arrest and growth inhibition (Fig. **2B**). Compared to the control group, FaDu cells treated with metformin exhibited an accumulation in the G2/M phase, increasing from 12.45±0.35 to 21.49±0.15. Concurrently, there was a decline in the G0/G1 peak after 48 hours, and the G2/M ratio increased with the rise in metformin concentration. These findings suggest that metformin impedes cell growth by obstructing progression through the G2/M phase (Fig. **2D** and Table **S1**).

### 3.3 Metformin Inhibits the Development of FaDu Cells, Possibly through the EMT Pathway

To investigate the molecular mechanisms underlying metformin's suppressive effects on tumor cell growth and metastasis, we performed Western blot analysis targeting key epithelial-mesenchymal transition (EMT) markers and Wnt pathway components. E-cadherin and N-cadherin, pivotal regulators of EMT, and β-catenin, the central mediator of Wnt signaling, were quantitatively assessed.

FaDu cells were treated with 5 mM and 10 mM metformin for 48 hours. β-catenin levels exhibited a non-significant increase at 5 mM metformin (*p* > 0.05), whereas a statistically significant reduction was observed at 10 mM (*p*< 0.05). Concurrently, E-cadherin protein expression showed a dose-dependent upregulation, with significant elevation in both treatment groups compared to controls (*p* < 0.001). In contrast, N-cadherin expression was markedly suppressed in the 10 mM metformin group (*p* < 0.01), indicating a metformin-induced reversal of EMT progression (Fig. **2E**). The quantitative analysis of β-catenin, E-cadherin, and N-cadherin protein expression levels is presented in Fig. (**2F**).

### 3.4 Metformin Suppressed Tumor Progression In Vivo

To further elucidate the effects of metformin on tumors *in vivo* and assess its potential for hepatotoxicity, we established xenograft models using nude mice. The study findings indicated that the metformin group exhibited lower tumor weights compared to the control group (Figs. **3A** and **3B**). Additionally, the metformin group showed a larger area of necrosis compared to the control group (Fig. **3C**, Tumor). The safety and toxicity of metformin were evaluated by measuring the body weight of the mice and conducting HE staining on the livers of the nude mice. The metformin-treated mice did not experience a notable decrease in body weight or show signs of hepatocellular necrosis when compared to the control group, suggesting the safety of metformin treatment (Fig. **3C**, Liver). Additionally, the immunohistochemical analysis conducted on tumor sections revealed that metformin significantly decreased Ki-67 expression, which is an indicator of tumor proliferation (Fig. **3D**). This finding further supports the conclusion that metformin inhibits the proliferation of HSCC cells *in vivo*.

To investigate how metformin influences autophagy in nude mice with transplanted tumors, TEM was employed to assess autophagic activity and mitochondrial architecture within the tumors. The findings from TEM indicated that there was a notable rise in the number of autophagosomes within the tumor tissues treated with metformin as opposed to the control group. In the control group, the mitochondria(M) displayed a slight swelling, with their membranes remaining largely intact. Conversely, in the metformin-treated group, many of the mitochondria (M) showed moderate swelling, characterized by distorted membranes and isolated vacuolar lesions (Fig. **3E**). These observations imply that metformin may hinder tumor progression, potentially by promoting autophagy *in vivo*.

### 3.5 RNA-seq Analysis of Differentially Expressed Genes and Pathway Enrichment in Metformin-treated Fadu Cells

The KEGG enrichment analysis revealed that FaDu cells treated with metformin showed significant enrichment in the ferroptosis, mitophagy, MAPK and NF-κB signaling pathways (Fig. **4A**). Additionally, the KEGG analysis suggested a notable reduction in the metformin-targeted FaDu cells regarding the cell cycle, DNA replication, mismatch repair, and base excision repair (Fig. **4B**). GO enrichment analysis revealed significant downregulation in biological processes related to DNA replication, cellular components of the nucleoplasm, and molecular functions involving protein binding (Fig. **4C**). RNA-seq analysis revealed that metformin led to the upregulation of 1,697 genes and the downregulation of 858 genes (Fig. **S4**). Transcriptome sequencing analysis revealed significant differential expression patterns, as illustrated in the heatmap (Fig. **4D**) and volcano plot (Fig. **4E**). Among the DEGs, PCAT1, SIK1, and SNHG12 showed elevated expression levels, while lncAROD exhibited reduced expression.

The results from the qPCR indicated a decline in lncAROD expression in both the metformin-treated cells and the transplanted tumors. In contrast, the expression level was found to be reduced in cells compared to that in the transplanted tumors (Fig. **4F**). The qPCR experiments were also performed to confirm the expression levels of several other genes identified in the RNA-seq analysis, including PCAT1, ZFAS1, MALAT1, and SIK1, all of which were notably upregulated in metformin-treated FaDu cells (Fig. **S1**).

### 3.6 Elevated Lncarod Expression in HNSCC Correlates with Clinical Survival Outcomes

To further investigate the expression of lncAROD in a large sample of clinical patient tissues, this study examined lncAROD expression in 302 HNSCC tissues and 44 adjacent non-cancerous tissues. The results indicated a significant increase in the expression of lncAROD within HNSCC tissues (***p<0.001, Fig. **5A**). Furthermore, an examination of 44 HNSCC samples alongside their adjacent normal tissues reinforced the elevated levels of lncAROD present in HNSCC tissues (***p<0.001, Fig. **5B**). ROC curve analysis was utilized to quantify the diagnostic performance of lncAROD measurements in distinguishing HNSCC cases from controls, establishing its clinical applicability. In this context, the X-axis illustrates the false positive rate, while the Y-axis denotes the true positive rate. The computed area under the curve (AUC) was found to be 0.839, indicating that lncAROD possesses significant sensitivity and specificity for HNSCC diagnosis (Fig. **5C**).

By integrating TCGA and GTEx datasets, we performed pan-cancer analysis of lncAROD expression patterns. Comparative assessment demonstrated significant upregulation of lncAROD in tumor samples relative to normal tissue counterparts across multiple malignancies. lncAROD exhibited significantly higher levels of expression in 19 of 33 cancer types, with the highest levels observed in HNSCC. This was closely followed by lung squamous cell carcinoma(LUSC) and esophageal cancer (ESCA) (Fig. **5D**).

Survival analysis employing the Kaplan-Meier methodology demonstrated the significant prognostic value of lncAROD expression in the patient cohort. Quantitative assessment revealed a 15.3-month decrease in median OS for high-expression patients(43 months) relative to low-expression counterparts(58.3 months), indicating lncAROD's potential role in HNSCC progression and adverse clinical outcomes (Fig. **5E**).

### 3.7 Knockdown of lncAROD Regulates FaDu Proliferative, Migration, and Invasion

To verify the previous finding that lncAROD participates in the regulation of HSCC, we transfected siRNA-lncAROD into FaDu cells. Following the observation of green fluorescence under a fluorescence microscope (Fig. **S2**), we further assessed the transfection efficiency of siRNA-lncAROD using RT-qPCR (Fig. **S3**). Colony formation assays demonstrated that siRNA-lncAROD-treated FaDu cells exhibited reduced proliferative capacity, though slightly higher than the metformin group. The metformin group combined with siRNA-lncAROD treatment yielded the fewest cells (Fig. **6A**, **B**). Additionally, the Transwell assay showed decreased migration and invasion of FaDu cells with low lncAROD expression (Fig. **6C**-**F**). These results indicate that lncAROD inhibits the proliferation, migration, and invasion of FaDu cells.

## 4 DISCUSSION

The treatment of HSCC is still in the exploratory phase due to the disease's complex etiology and the distinct circumstances of each patient [45]. Metformin has demonstrated an improvement in OS rates for patients with HNSCC [46, 47]. In this study, the CCK-8 assay, colony formation assay, and wound healing assay demonstrated that metformin significantly inhibited cell proliferation and migration. Furthermore, flow cytometric evaluation demonstrated significant increases in apoptotic cell populations and induction of cell cycle arrest, consistent with prior published findings in related research [48, 49]. Compared to the wound healing and CCK-8 assays, the colony formation assay showed that 10 mM metformin resulted in a more robust suppression of cell proliferation. This difference may result from the prolonged 2-week experimental duration, allowing for cumulative drug effects on cellular proliferation [20].

The study results of this research indicated that metformin affects several signaling pathways involved in tumor cell suppression, including reductions in DNA replication, RNA transport, ferroptosis and protein processing. Heatmap analysis of genes demonstrating significant expression changes revealed that metformin treatment upregulates the expression of PRKAB2, ULK1, CDH2, and CDH13, while downregulating MLYCD. All of these genes are known targets of the AMPK signaling pathway [50-52]. Metformin alters the expression levels of genes like MALAT1 and SIK1, which may be associated with metabolic processes [53, 54].

Bioinformatic analysis of RNA-seq data identified lncAROD as among the most markedly downregulated transcripts. This long non-coding RNA has been established as a crucial regulator in cancer pathogenesis, particularly in the progression of squamous cell carcinoma [55, 56]. Its upregulation has been linked to unfavorable prognosis and malignant tumor development across a range of cancers, such as hepatocellular carcinoma (HCC) and HNSCC [56, 57]. The irregular expression of lncAROD has been associated with various types of cancer, emphasizing its possible role as a diagnostic and prognostic indicator in the management of cancer [58, 59].

The PCR was extracted from FaDu cells as well as tumor samples from nude mice, and qRT-PCR experiments revealed a high expression of lncAROD in both FaDu cells and tumor tissue; however, this expression decreased following treatment with metformin. These findings indicate that metformin might impede tumor growth by lowering lncAROD levels. Research has identified lncAROD as a significant contributor to HNSCC development, where it orchestrates tumor progression through multifaceted mechanisms involving protein complex formation and modulation of energy metabolism pathways such as aerobic glycolysis [56]. Silencing of lncAROD in esophageal squamous cell carcinoma led to a decrease in tumor growth and enhanced sensitivity to radiation [55]. Understanding how lncAROD affects cancer development will provide insights into potential therapeutic targets and diagnostic approaches for cancer treatment [60, 61].

Epithelial-mesenchymal transition (EMT) is essential for the process of cancer metastasis and is associated with significant epigenetic alterations [62-64]. Reduced levels of E-cadherin in HNSCC are strongly linked to a lower OS rate [65, 66]. WB suggested that metformin has potential in suppressing the EMT by influencing its key markers, including E-cadherin, β-catenin, and N-cadherin. The increase of E-cadherin inhibits tumor metastasis through various downstream transcriptional pathways [67], while the decrease of N-cadherin and β-catenin also inhibits tumor cell metastasis [68, 69]. Metformin may inhibit the growth of Fadu cells by targeting the EMT pathway. lncAROD serves as the lncRNA-activating regulator of DKK1 [70], which is known to function as an extracellular inhibitor of the Wnt/β-catenin pathway [71, 72]. The reduction of lncAROD induced by metformin could also lead to the inactivation of the Wnt pathway, thereby further inhibiting the EMT pathway [73, 74]. Consequently, we hypothesize that lncAROD may serve as a potential target for metformin.

Furthermore, the *in vivo* xenograft model provided additional confirmation of the tumor-suppressive properties of metformin. In the nude mice transplanted with tumors, the internal organs appeared normal, and histological examination of the liver through HE staining showed no signs of liver damage. Moreover, both HE staining and Ki67 analysis of the tumors demonstrated a notable increase in apoptosis within the metformin-treated group. Ki67 is an antigen associated with cell proliferation [75]. Ki-67 positivity generally indicates an increased rate of cell proliferation, a higher degree of tumor malignancy, and a worse prognosis [76]. Nevertheless, the outcomes from HE staining and TEM of the tumor did not show a definitive trend towards tumor growth inhibition, as highlighted by Ki67. These results imply that the suppression of tumor cells *in vivo* is likely mediated by a blend of various mechanisms.

The findings indicate that several factors influence the impact of metformin on HSCC.Despite these findings, the specific mechanisms by which metformin acts on HSCC remain largely unexplored. Consequently, additional research is required to clarify the drug's mechanism of action, particularly through *in vivo* experiments and various functional analyses, in order to identify how lncAROD facilitates the advancement of HSCC accurately.

## CONCLUSION

The findings indicate that several factors influence the impact of metformin on HSCC.Despite these findings, the specific mechanisms by which metformin acts on HSCC remain largely unexplored. Consequently, additional research is re- quired to clarify the drug's mechanism of action, particularly through *in vivo* experiments and various functional analyses, in order to identify how lncAROD facilitates the advancement of HSCC accurately.

## AUTHORS' CONTRIBUTIONS

The authors confirm their contribution to the paper as follows: L conceptualized and led the project, wrote the original version of the manuscript, and revised it. HX performed the pathological experiments and analyzed the data. ML conducted the molecular experiments and analyzed the data. DW conceptualized and led the project, provided funding, and revised the manuscript. All authors contributed to the revision and editing of the final manuscript.

## Figures and Tables

**Fig. (1):**
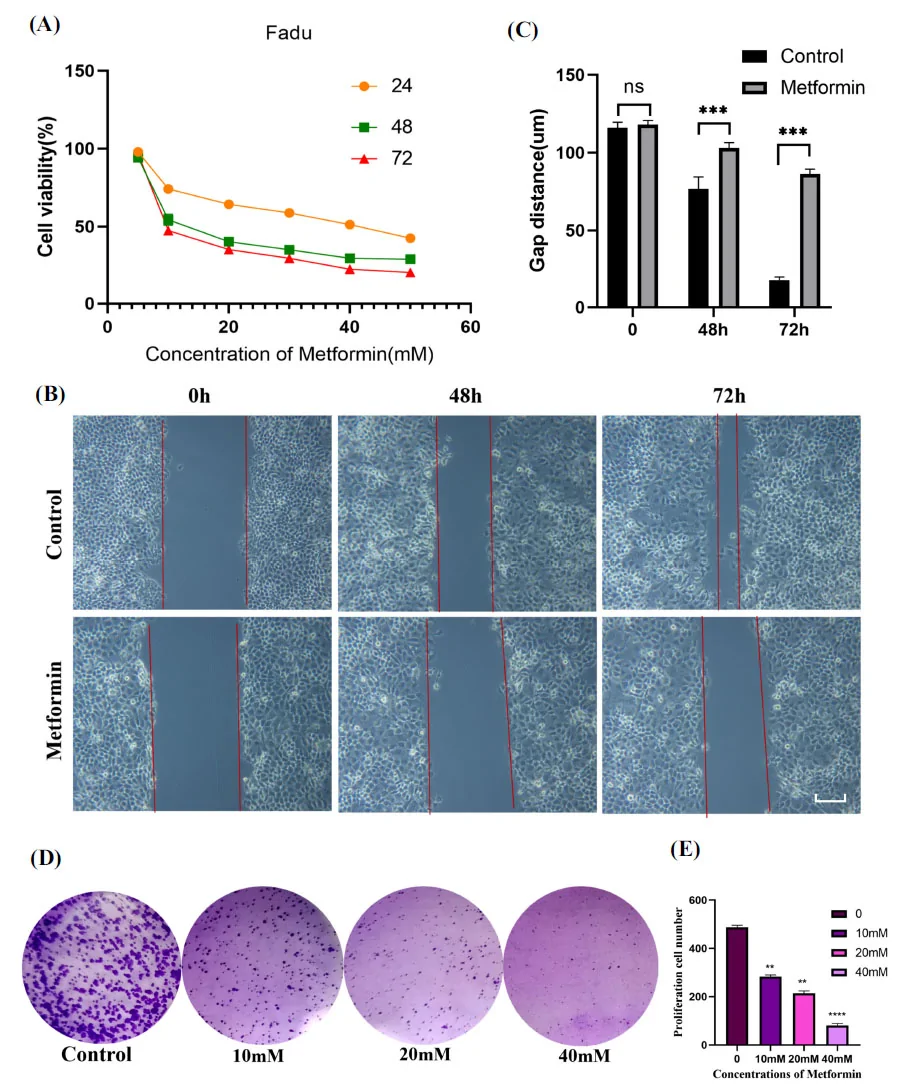
Metformin suppressed the proliferation of FaDu cells. (**A**) Cell viability of FaDu cells was assessed by CCK-8 assay after treated by metformin,over a period of 24,48 and 72 h. (**B**) Representative images of wound-healing assay,at 0, 48, and 72 h,Scale bar: 150 µm.(**C**) The distances of relative migration were analyzed in the wound-healing assay. (**D**) The proliferative activity of FaDu cells was examined using the colony formation assay. (**E**) A quantitative analysis was conducted on FaDu cells proliferating at different concentrations of metformin.

**Fig. (2):**
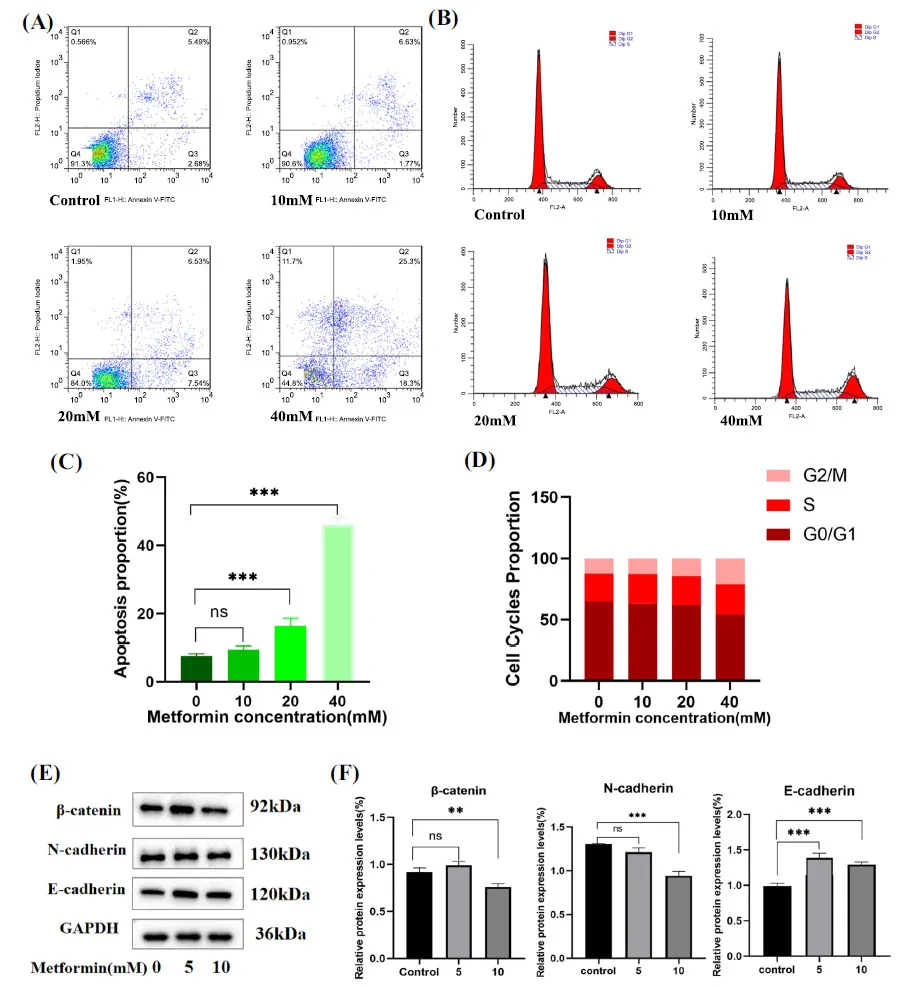
Effect of Metformin on the apoptosis and cell cycle of FaDu cells,and its relationship with EMT pathway. (**A**) Flow cytometry analysis of apoptosis in FaDu cells treated with varying concentrations of metformin (0, 10 mM, 20 mM, 40 mM). (**B**) Cell cycle analysis of FaDu cells exposed to different metformin concentrations (0, 10 mM, 20 mM, 40 mM) for 48 hours (n = 3) was performed. (**C**) The percentage of FaDu cells in total apoptosis upon treatment of metformin. (**D**) The percentage of cell cycle distribution in metformin-treated FaDu cells. (**E**) The protein levels of β-catenin、N-cadherin and E-cadherin in FaDu cells pretreated with metformin for 48 hours were evaluated using WB. (**F**) The quantification of the results from WB is presented.

**Fig. (3):**
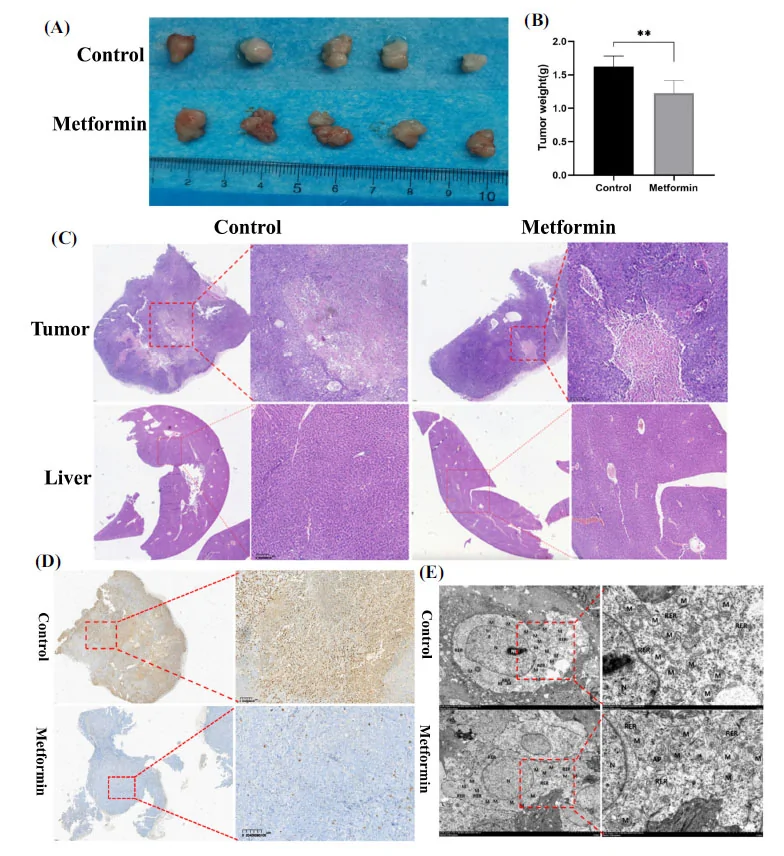
Metformin inhibited the proliferation and induced apoptosis in FaDu cells *in vivo*. (**A**) The tumors were excised following the euthanasia of all mice. (**B**) Tumor weight measurement post metformin administration. (**C**) H&E staining was performed to evaluate the histological characteristics of both the tumor and liver. (**D**) Immunohistochemical analysis was conducted to observe the expression of Ki-67 within the tumor. (**E**) TEM illustrating metformin-triggered apoptosis and autophagy *in vivo*. Scale bar: 100 µm.

**Fig. (4):**
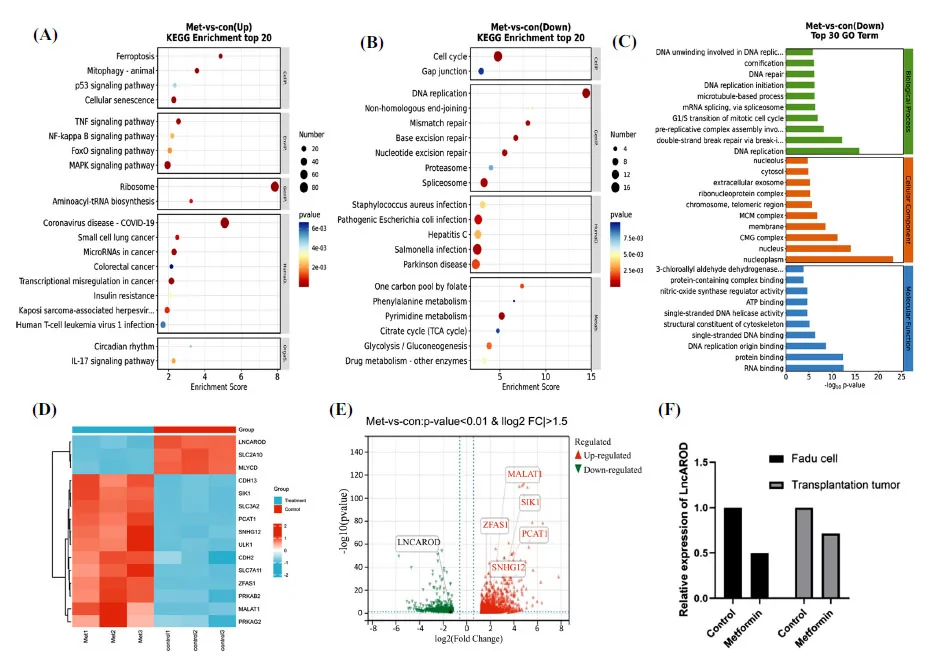
Transcriptome sequencing revealed metformin in FaDu cells. (**A**) GO pathway enrichment KEGG enrichment analyses of up 20. (**B**) KEGG enrichment analyses of down 20. (**C**) GO enrichment analysis of top 30. (**D**) The bar graph illustrates genes analyzed through RNA-seq. (**E**) The volcano plots of DEG. (**F**) qRT-PCR was employed to measure lncAROD transcript levels in both metformin-treated cellular models and xenograft tumor tissues.

**Fig. (5):**
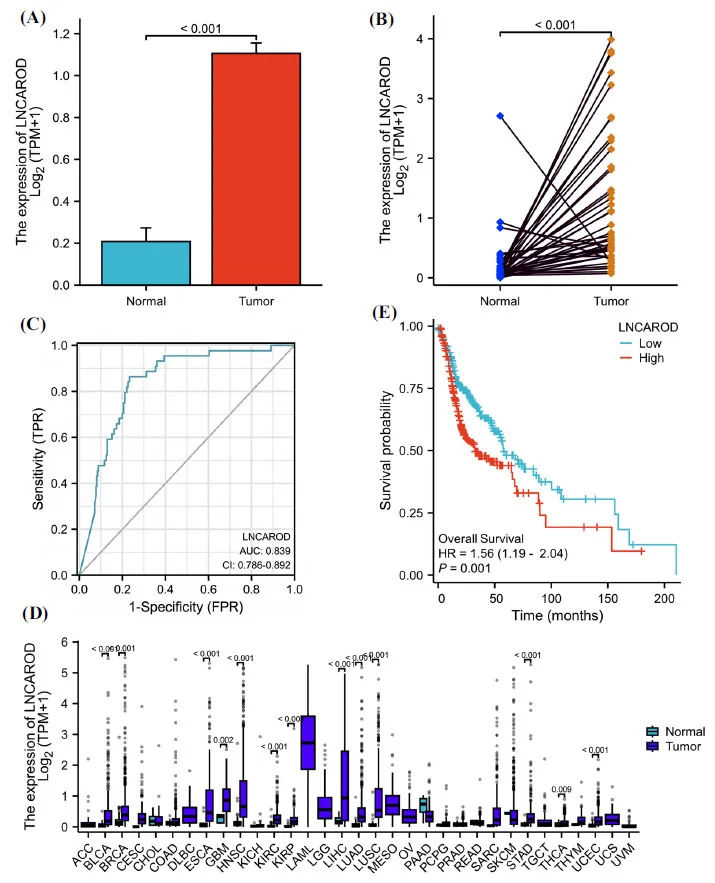
Bioinformatics analysis and survival analysis. (**A**) The lncAROD expression levels in clinical tissues of HNSCC. (**B**) lncAROD expression in 44 pairs of tumors and adjacent non-tumor tissues. (**C**) ROC curve analysis. (**D**) lncAROD expression in various tumor types. (**E**) The Kaplan–Meier survival analysis associated with lncAROD expression.

**Fig. (6):**
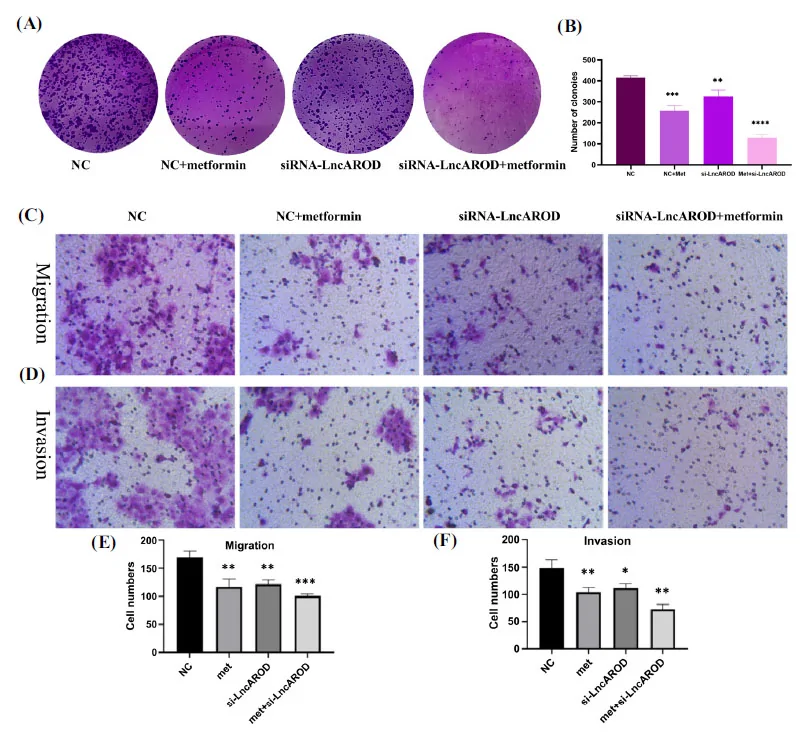
Knockdown of lncAROD inhibits the malignant malignant characteristics of HSCC. (**A** and **B**) Colony formation assays demonstrated the proliferation of FaDu cells. (**C**) Transwell cell migration assays analysis of FaDu cells. (**D**) Transwell cell invasion assays analysis of FaDu cells. (**E**) The number of migrated cells observed per field. (**F**) The count of invaded cells per field.

## Data Availability

The datasets generated and analyzed during the current study are available in the TCGA repository for HNSC (TCGA-HNSC). The GTEx datasets used for the analyses described in this manuscript were obtained from dbGaP. Further information is available from the corresponding authors upon reasonable request.

## LIST OF ABBREVIATIONS

AUC: Area Under the Curve
EMT: Epithelial-Mesenchymal Transition
ESCA: Esophageal Cancer
FBS: Fetal Bovine Serum
GO: Gene Ontology
GSEA: Gene Set Enrichment Analysis
H&E: Hematoxylin‒Eosin
HCC: Hepatocellular Carcinoma
HNSCC: Head and Neck Squamous Cell Carcinoma
HSCC: Hypopharyngeal Squamous Cell Carcinoma
IHC: Immunohistochemical
KEGG: Kyoto Encyclopedia of Genes and Genomes
LUSC: Lung Squamous Cell Carcinoma
OS: Overall Survival
PBS: Phosphate-Buffered Saline
PI: Propidium Iodide
qRT-PCR: Quantitative Real-time PCR
RNA-seq: RNA Sequencing
SD: Standard Deviation
TCGA: The Cancer Genome Atlas Program
TEM: Transmission Electron Microscopy
TME: Tumor Microenvironment

## References

[1] Jakstas T., Bartnykaite A., Padervinskis E., Vegiene A., Juozaityte E., Uloza V., Ugenskiene R. (2024). The association of TP53, BCL2, BAX and NOXA SNPs and laryngeal squamous cell carcinoma development.. Int. J. Mol. Sci..

[2] Cai Z., Chen L., Chen S., Fang R., Chen X., Lei W. (2023). Single-cell RNA sequencing reveals pro-invasive cancer-associated fibroblasts in hypopharyngeal squamous cell carcinoma.. Cell. Commun. Signal..

[3] Luo X., Huang X., Liu S., Wang X., Luo J., Xiao J., Wang K., Qu Y., Chen X., Zhang Y., Wang J., Zhang J., Xu G., Gao L., Wu R., Yi J. (2022). Response-Adapted treatment following radiotherapy in patients with resectable locally advanced hypopharyngeal carcinoma.. JAMA Netw Open.

[4] Visini M., Giger R., Shelan M., Elicin O., Anschuetz L. (2021). Predicting factors for oncological and functional outcome in hypopharyngeal cancer.. Laryngoscope.

[5] Tassler A.B., Gooding W.E., Ferris R.L. (2019). Hypopharyngeal cancer treatment: Does initial surgery confer survival benefit?. Head Neck.

[6] Kwon D.I., Miles B.A. (2019). Hypopharyngeal carcinoma: Do you know your guidelines?. Head Neck.

[7] Solomon B., Young R.J., Rischin D. (2018). Head and neck squamous cell carcinoma: Genomics and emerging biomarkers for immunomodulatory cancer treatments.. Semin Cancer Biol..

[8] Desai N., Divatia M.K., Jadhav A., Wagh A. (2023). Aggressive cutaneous squamous cell carcinoma of the head and neck: A review.. Curr. Oncol..

[9] Panda S., Sakthivel P., Gurusamy K.S., Sharma A., Thakar A. (2022). Treatment options for resectable hypopharyngeal squamous cell carcinoma: A systematic review and meta-analysis of randomized controlled trials.. PLoS One.

[10] Ruffin A.T., Li H., Vujanovic L., Zandberg D.P., Ferris R.L., Bruno T.C. (2023). Improving head and neck cancer therapies by immunomodulation of the tumour microenvironment.. Nat. Rev. Cancer.

[11] Xia Y., Sun M., Huang H., Jin W.L. (2024). Drug repurposing for cancer therapy.. Signal. Transduct. Target Ther..

[12] Singhal S., Maheshwari P., Krishnamurthy P.T., Patil V.M. (2022). Drug repurposing strategies for non-cancer to cancer therapeutics.. Anticancer Agents Med. Chem..

[13] Jin M.Z., Jin W.L. (2020). The updated landscape of tumor microenvironment and drug repurposing.. Signal. Transduct. Target Ther..

[14] Fu Z., Zhang X., Gao Y., Fan J., Gao Q. (2023). Enhancing the anticancer immune response with the assistance of drug repurposing and delivery systems.. Clin. Transl. Med..

[15] Jin M., Zeng B., Liu Y., Jin L., Hou Y., Liu C., Liu W., Wu H., Chen L., Gao Z., Huang W. (2022). Co-delivery of repurposing itraconazole and vegf sirna by composite nanoparticulate system for collaborative anti-angiogenesis and anti-tumor efficacy against breast cancer.. Pharmaceutics.

[16] Fayyaz S., Atia-Tul-Wahab, Irshad R., Siddiqui R.A., Choudhary M.I. (2024). Antidepressant sertraline hydrochloride inhibits the growth of HER2+ AU565 breast cancer cell line through induction of apoptosis and cell cycle arrest.. Anticancer Agents Med. Chem..

[17] Anwer M.S., Abdel-Rasol M.A., El-Sayed W.M. (2025). Emerging therapeutic strategies in glioblastsoma: Drug repurposing, mechanisms of resistance, precision medicine, and technological innovations.. Clin. Exp. Med..

[18] Wang Y., Yang Y., Chen S., Wang J. (2021). DeepDRK: A deep learning framework for drug repurposing through kernel-based multi-omics integration.. Brief Bioinform..

[19] Abdel-Wahab A.F., Mahmoud W., Al-Harizy R.M. (2019). Targeting glucose metabolism to suppress cancer progression: Prospective of anti-glycolytic cancer therapy.. Pharmacol. Res..

[20] Foretz M., Guigas B., Viollet B. (2023). Metformin: Update on mechanisms of action and repurposing potential.. Nat. Rev. Endocrinol..

[21] Coyle C., Cafferty F.H., Vale C., Langley R.E. (2016). Metformin as an adjuvant treatment for cancer: A systematic review and meta-analysis.. Ann Oncol..

[22] Sun Y., Cheng J., Nie D., Fang Q., Li C., Zhang Y. (2024). Metformin inhibits cell proliferation and ACTH secretion in AtT20 cells via regulating the MAPK pathway.. Mol. Cell. Endocrinol..

[23] Rêgo D.F., Elias S.T., Amato A.A., Canto G.D.L., Guerra E.N.S. (2017). Anti-tumor effects of metformin on head and neck carcinoma cell lines: A systematic review.. Oncol. Lett..

[24] Vasan K., Werner M., Chandel N.S. (2020). Mitochondrial metabolism as a target for cancer therapy.. Cell. Metab..

[25] Chen Y.H., Yang S.F., Yang C.K., Tsai H.D., Chen T.H., Chou M.C., Hsiao Y.H. (2020). Metformin induces apoptosis and inhibits migration by activating the AMPK/p53 axis and suppressing PI3K/AKT signaling in human cervical cancer cells.. Mol. Med. Rep.

[26] Yang J., Zhou Y., Xie S., Wang J., Li Z., Chen L., Mao M., Chen C., Huang A., Chen Y., Zhang X., Khan N.U.H., Wang L., Zhou J. (2021). Metformin induces Ferroptosis by inhibiting UFMylation of SLC7A11 in breast cancer.. J. Exp. Clin. Cancer Res..

[27] Padilla J., Lee J. (2021). A novel therapeutic target, BACH1, regulates cancer metabolism.. Cells.

[28] Huang X., Sun T., Wang J., Hong X., Chen H., Yan T., Zhou C., Sun D., Yang C., Yu T., Su W., Du W., Xiong H. (2023). Metformin reprograms tryptophan metabolism to stimulate CD8+ T-cell function in colorectal cancer.. Cancer Res..

[29] Morale M.G., Tamura R.E., Rubio I.G.S. (2022). Metformin and cancer hallmarks: Molecular mechanisms in thyroid, prostate and head and neck cancer models.. Biomolecules.

[30] Triggle C.R., Mohammed I., Bshesh K., Marei I., Ye K., Ding H., MacDonald R., Hollenberg M.D., Hill M.A. (2022). Metformin: Is it a drug for all reasons and diseases?. Metabolism.

[31] Tufail M., Jiang C.H., Li N. (2024). Altered metabolism in cancer: Insights into energy pathways and therapeutic targets.. Mol. Cancer.

[32] Halma M., Tuszynski J., Marik P. (2023). Cancer metabolism as a therapeutic target and review of interventions.. Nutrients.

[33] Kroemer G., Pouyssegur J. (2008). Tumor cell metabolism: Cancer’s Achilles’ heel.. Cancer Cell..

[34] Tan Y.T., Lin J.F., Li T., Li J.J., Xu R.H., Ju H.Q. (2021). LncRNA-mediated posttranslational modifications and reprogramming of energy metabolism in cancer.. Cancer Commun..

[35] Zhou H., Hao X., Zhang P., He S. (2023). Noncoding RNA mutations in cancer.. Wiley Interdiscip Rev. RNA.

[36] Ahmad M., Weiswald L.B., Poulain L., Denoyelle C., Meryet-Figuiere M. (2023). Involvement of lncRNAs in cancer cells migration, invasion and metastasis: Cytoskeleton and ECM crosstalk.. J. Exp. Clin. Cancer Res..

[37] Li D., Feng J., Wu T., Wang Y., Sun Y., Ren J., Liu M. (2013). Long intergenic noncoding RNA HOTAIR is overexpressed and regulates PTEN methylation in laryngeal squamous cell carcinoma.. Am J. Pathol..

[38] Xu E., Liang X., Ji Z., Zhao S., Li L., Lang J. (2020). Blocking long noncoding RNA MALAT1 restrained the development of laryngeal and hypopharyngeal carcinoma.. Eur Arch. Otorhinolaryngol..

[39] Garcia-Padilla C., Lozano-Velasco E., Muñoz-Gallardo M.M., Castillo-Casas J.M., Caño-Carrillo S., Martínez-Amaro F.J., García-López V., Aránega A., Franco D., García-Martínez V., López-Sánchez C. (2022). LncRNA H19 impairs chemo and radiotherapy in tumorigenesis.. Int. J. Mol. Sci..

[40] Lin D., Shen L., Luo M., Zhang K., Li J., Yang Q., Zhu F., Zhou D., Zheng S., Chen Y., Zhou J. (2021). Circulating tumor cells: Biology and clinical significance.. Signal. Transduct. Target Ther..

[41] Zhang H., Lu X., Lu B., Gullo G., Chen L. (2024). Measuring the composition of the tumor microenvironment with transcriptome analysis: past, present and future.. Future Oncol..

[42] Mollaei M., Hassan Z.M., Khorshidi F., Langroudi L. (2021). Chemotherapeutic drugs: Cell death- and resistance-related signaling pathways. Are they really as smart as the tumor cells?. Transl. Oncol..

[43] Zheng D., Shen Y., Wei Z., Wan X., Xie M., Yao H., Wang Z. (2021). Transcriptome sequencing reveals a lncRNA–mRNA interaction network in extramammary Paget’s disease.. BMC Med. Genomics.

[44] Tian W., Zhou J., Chen M., Qiu L., Li Y., Zhang W., Guo R., Lei N., Chang L. (2022). Bioinformatics analysis of the role of aldolase A in tumor prognosis and immunity.. Sci. Rep.

[45] Nuth M., Benakanakere M., Ricciardi R. (2022). Discovery of a potent cytotoxic agent that promotes G 2 /M phase cell cycle arrest and apoptosis in a malignant human pharyngeal squamous carcinoma cell line.. Int. J. Oncol..

[46] Rêgo D.F., Pavan L.M.C., Elias S.T., De Luca Canto G., Guerra E.N.S. (2015). Effects of metformin on head and neck cancer: A systematic review.. Oral Oncol..

[47] Wu X., Yeerna H., Goto Y., Ando T., Wu V.H., Zhang X., Wang Z., Amornphimoltham P., Murphy A.N., Tamayo P., Chen Q., Lippman S.M., Gutkind J.S. (2019). Metformin inhibits progression of head and neck squamous cell carcinoma by acting directly on carcinoma-initiating cells.. Cancer Res..

[48] Di Matteo S., Nevi L., Overi D., Landolina N., Faccioli J., Giulitti F., Napoletano C., Oddi A., Marziani A.M., Costantini D. (2021). Metformin exerts anti-cancerogenic effects and reverses epithelial-to-mesenchymal transition trait in primary human intrahepatic cholangiocarcinoma cells.. Sci. Rep.

[49] Nguyen M.T., Choe H.C., Kim B.H., Ahn S.G. (2022). A new link between apoptosis induced by the metformin derivative HL156A and autophagy in oral squamous cell carcinoma.. Eur. J. Pharmacol..

[50] Xavier A.E.T., Veronez L.C., Nagano L.F.P., Correa C.A.P., Baroni M., Ramos M.S., Queiroz R.G.P., Fernandes Molina C.A., Yunes J.A., Brandalise S.R., Antonini S.A.R., Tone L.G., Valera E.T., Scrideli C.A. (2024). Low PRKAB2 expression Is Associated with Poor Outcomes in Pediatric Adrenocortical Tumors, and Treatment with Rottlerin Increases the PRKAB2 Level and Inhibits Tumorigenic Aspects in the NCI-H295R Adrenocortical Cancer Cell Line.. Cancers.

[51] Wu J., Zhang D., Li J., Deng X., Liang G., Long Y., He X., Dai T., Ren D. (2017). MACC1 induces autophagy to regulate proliferation, apoptosis, migration and invasion of squamous cell carcinoma.. Oncol. Rep.

[52] Xue Q., Kang R., Klionsky D.J., Tang D., Liu J., Chen X. (2023). Copper metabolism in cell death and autophagy.. Autophagy.

[53] Safavi K., Abedpoor N., Hajibabaie F., Ka E. (2025). Mitigating diabetic cardiomyopathy: The synergistic potential of sea buckthorn and metformin explored via bioinformatics and chemoinformatics.. Biology.

[54] Liao Q., Xu W., Luo Q., Wen X. (2021). Zhenqing recipe relieves diabetic nephropathy through the SIK1/SREBP-1c axis in type 2 diabetic rats.. Am J. Transl. Res..

[55] Shi X., Zhang X., Huang X., Zhang R., Pan S., Huang S., Wang Y., Ke Y., Guo W., Liu X., Hao Y., Li Y., Zhao X., Sun Y., Li J., Ma H., Zhao X. (2024). N6-methyladenosine-mediated upregulation of LNCAROD confers radioresistance in esophageal squamous cell carcinoma through stabilizing PARP1.. Clin. Transl. Med..

[56] Ban Y., Tan P., Cai J., Li J., Hu M., Zhou Y., Mei Y., Tan Y., Li X., Zeng Z., Xiong W., Li G., Li X., Yi M., Xiang B. (2020). LNCAROD is stabilized by m6A methylation and promotes cancer progression via forming a ternary complex with HSPA1A and YBX1 in head and neck squamous cell carcinoma.. Mol. Oncol..

[57] Tang J., Zhang J., Lu Y., He J., Wang H., Liu B., Tu C., Li Z. (2023). Novel insights into the multifaceted roles of m6A-modified LncRNAs in cancers: biological functions and therapeutic applications.. Biomark Res..

[58] Cerk S., Schwarzenbacher D., Adiprasito J., Stotz M., Hutterer G., Gerger A., Ling H., Calin G., Pichler M. (2016). Current status of long non-coding RNAs in 0.. Int. J. Mol. Sci..

[59] Qian Y., Shi L., Luo Z. (2020). Long Non-coding RNAs in cancer: Implications for diagnosis, prognosis, and therapy.. Front. Med..

[60] Ansari S., Nikpour P. (2023). LNCAROD promotes the proliferation and migration of gastric cancer: a bioinformatics analysis and experimental validation.. Funct. Integr. Genomics.

[61] Toden S., Zumwalt T.J., Goel A. (2021). Non-coding RNAs and potential therapeutic targeting in cancer.. Biochim Biophys. Acta. Rev. Cancer.

[62] Dong Y., Hu H., Zhang X., Zhang Y., Sun X., Wang H., Kan W., Tan M., Shi H., Zang Y., Li J. (2023). Phosphorylation of PHF2 by AMPK releases the repressive H3K9me2 and inhibits cancer metastasis.. Signal. Transduct. Target Ther..

[63] Allgayer H., Mahapatra S., Mishra B., Swain B., Saha S., Khanra S., Kumari K., Panda V.K., Malhotra D., Patil N.S., Leupold J.H., Kundu G.C. (2025). Epithelial-to-mesenchymal transition (EMT) and cancer metastasis: The status quo of methods and experimental models 2025.. Mol. Cancer.

[64] Dong B., Qiu Z., Wu Y. (2020). Tackle epithelial-mesenchymal transition with epigenetic drugs in cancer.. Front. Pharmacol..

[65] Dumitru C.S., Ceausu A.R., Comsa S., Raica M. (2022). Loss of E-Cadherin expression correlates with ki-67 in head and neck squamous cell carcinoma.. In Vivo.

[66] Yang H.L., Chang C.W., Vadivalagan C., Pandey S., Chen S.J., Lee C.C., Hseu J.H., Hseu Y.C. (2024). Coenzyme Q0 inhibited the NLRP3 inflammasome, metastasis/EMT, and Warburg effect by suppressing hypoxia-induced HIF-1α expression in HNSCC cells.. Int. J. Biol. Sci..

[67] Na T.Y., Schecterson L., Mendonsa A.M., Gumbiner B.M. (2020). The functional activity of E-cadherin controls tumor cell metastasis at multiple steps.. Proc. Natl. Acad. Sci. USA.

[68] Loh C.Y., Chai J., Tang T., Wong W., Sethi G., Shanmugam M., Chong P., Looi C. (2019). The e-cadherin and n-cadherin switch in epithelial-to-mesenchymal transition: Signaling, therapeutic implications, and challenges.. Cells.

[69] Gao S., Hu J., Wu X., Liang Z. (2018). PMA treated THP-1-derived-IL-6 promotes EMT of SW48 through STAT3/ERK-dependent activation of Wnt/β-catenin signaling pathway.. Biomed. Pharmacother..

[70] Ntini E., Louloupi A., Liz J., Muino J.M., Marsico A., Ørom U.A.V. (2018). Long ncRNA A-ROD activates its target gene DKK1 at its release from chromatin.. Nat. Commun..

[71] Liang L., Tu Y., Lu J., Wang P., Guo Z., Wang Q., Guo K., Lan R., Li H., Liu P. (2019). Dkk1 exacerbates doxorubicin-induced cardiotoxicity by inhibiting the Wnt/β-catenin signaling pathway.. J. Cell. Sci..

[72] Jiang H., Zhang Z., Yu Y., Chu H.Y., Yu S., Yao S., Zhang G., Zhang B.T. (2022). Drug discovery of DKK1 inhibitors.. Front. Pharmacol..

[73] Xue W., Yang L., Chen C., Ashrafizadeh M., Tian Y., Sun R. (2024). Wnt/β-catenin-driven EMT regulation in human cancers.. Cell. Mol. Life. Sci..

[74] Bai Y., Sha J., Kanno T. (2020). The role of carcinogenesis-related biomarkers in the wnt pathway and their effects on epithelial–mesenchymal transition (EMT) in oral squamous cell carcinoma.. Cancers.

[75] Zhou G., Wu H., Lin J., Lin R., Feng B., Liu Z. (2021). TRIM21 is decreased in colitis-associated cancer and negatively regulates epithelial carcinogenesis.. Inflamm. Bowel Dis..

[76] Zhang T., Pabla S., Lenzo F.L., Conroy J.M., Nesline M.K., Glenn S.T., Papanicolau-Sengos A., Burgher B., Giamo V., Andreas J., Wang Y., Bshara W., Madden K.G., Shirai K., Dragnev K., Tafe L.J., Gupta R., Zhu J., Labriola M., McCall S., George D.J., Ghatalia P., Dayyani F., Edwards R., Park M.S., Singh R., Jacob R., George S., Xu B., Zibelman M., Kurzrock R., Morrison C. (2020). Proliferative potential and response to nivolumab in clear cell renal cell carcinoma patients.. OncoImmunology.

