# Supplementary material for: Metformin Inhibits the Growth of Hypopharyngeal Squamous Cell Carcinoma of Fadu Cell and Down-Regulates LncAROD to Improve Prognosis
Source: Anticancer Agents Med Chem. 2025 Oct 29;26(11):1079–92. doi: 10.2174/0118715206427268251006112304 (PMC13673442; doi:10.2174/0118715206427268251006112304)
Supplement: Supplementary file 1 [file ACAMC-26-11-1079_SD1.pdf]

SUPPLEMENTARY MATERIAL

Metformin Inhibits the Growth of Hypopharyngeal Squamous Cell Carcinoma of Fadu Cell and Down-Regulates LncAROD to Improve Prognosis

Fang Liu[1,2], Huihui Xie[3], Meiyu Liu[4] and Desheng Wang[5,*]

[1]Fujian Medical University Union Hospital, Fuzhou, 350001, China; [2]Department of Otorhinolaryngology Head and Neck Surgery, First Affiliated Hospital of Gannan Medical University, Ganzhou, 341000, China; [3]Department of Pathology, First Affiliated Hospital of Gannan Medical University, Ganzhou, 341000, China; [4]Gannan Medical University, Ganzhou, 341000, China; [5]Department of Otolaryngology, Fujian Medical University Union Hospital, Fuzhou, 350001, China

Table S1. Effect of metformin on the cell cycle of FaDu cells.

	G0/G1(%)	S(%)	G2/M(%)
0mM	64.12±0.85	23.43±0.56	12.45±0.35
10mM	63.99±0.53	24.14±0.65	11.87±0.20
20mM	62.95±0.90	23.40±0.60	13.66±0.76
40mM	54.16±0.25	24.35±0.60	21.49±0.15

Table S2. The primers used in the study.

Gene Name		
LncAROD	forward	5’-CCACAACGGCAACCAGTAAA-3’
	reverse	5’-AGGCGTTCCACCTGCAAATA-3’
GAPDH	forward	5’-CAGGAGGCATTGCTGATGAT-3’
	reverse	5’-GAAGGCTGGGGCTCATTT-3’
MALAT1	forward	5’-GCTCTGTGGTGTGGGATTGA -3’
	reverse	5’-GTGGCAAAATGGCGGACTTT-3’
ZFAS1	forward	5’- ATACGCTATTGTCCTGCCCG -3’
	reverse	5’- TCCATCGCCAGTTTTCGTCA -3’
SIK1	forward	5’- CAGCTACACCTCCAACCTGG -3’
	reverse	5’- TTTTGCAGTGACTCCACCGT -3’
PCAT1	forward	5’-TGTGCCTCTAAGTGCCAGTG-3’
	reverse	5’-TGTATCTGCGCACCCTTTGA-3’

Table S3. siRNA sequences.

Gene	Sense (5′-3′)	Antisense (5′-3′)
Negative control	UUCUCCGAACGUGUCACGUTT	ACGUGACACGUUCGGAGAATT
LncAROD siRNA	CUCUAAUUGACCGAGACAATT	UUGUCUCGGUCAAUUAGAGTT

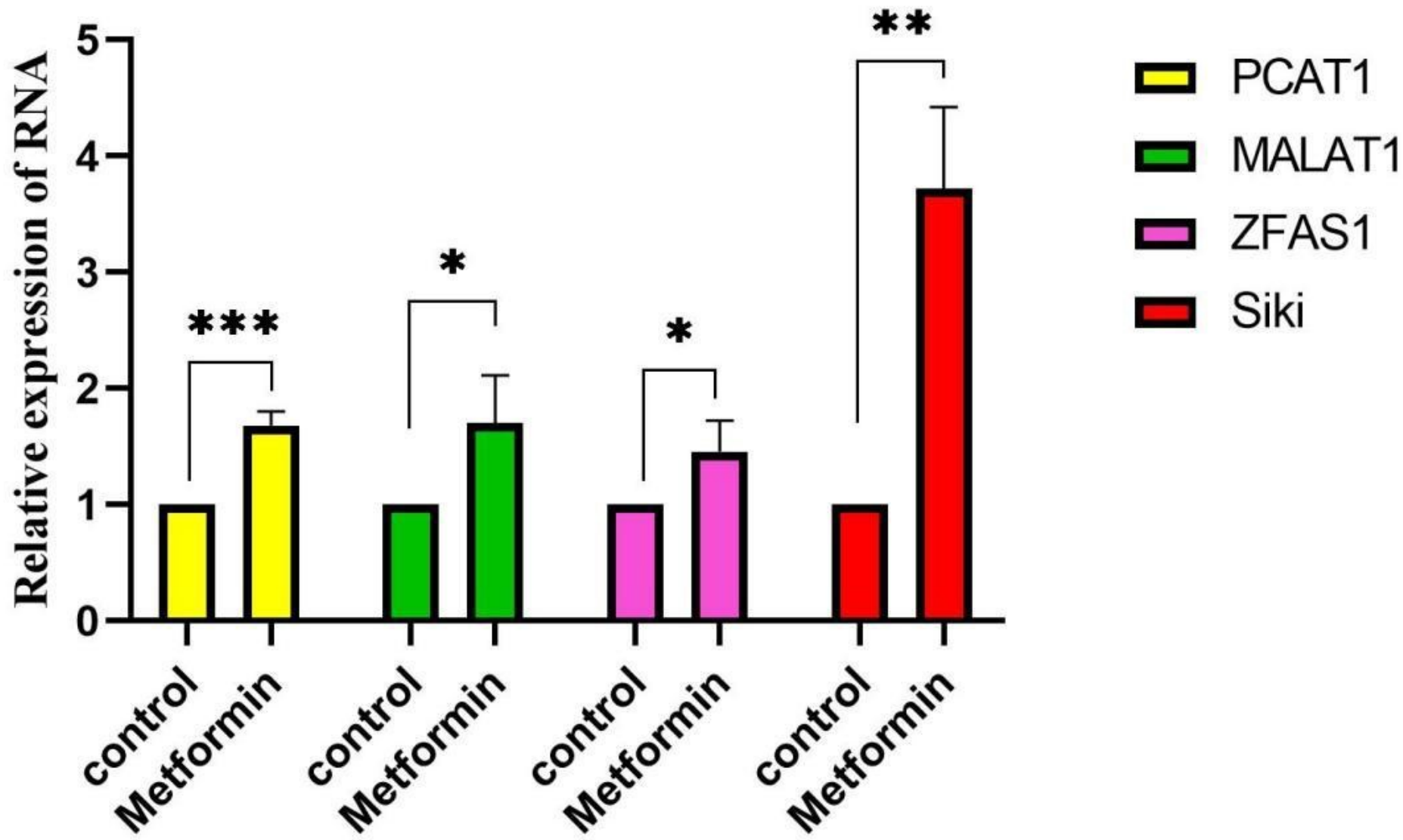

Figure S1 The RT-qPCR detection of other RNA expression in metformin-treated FaDu cells.

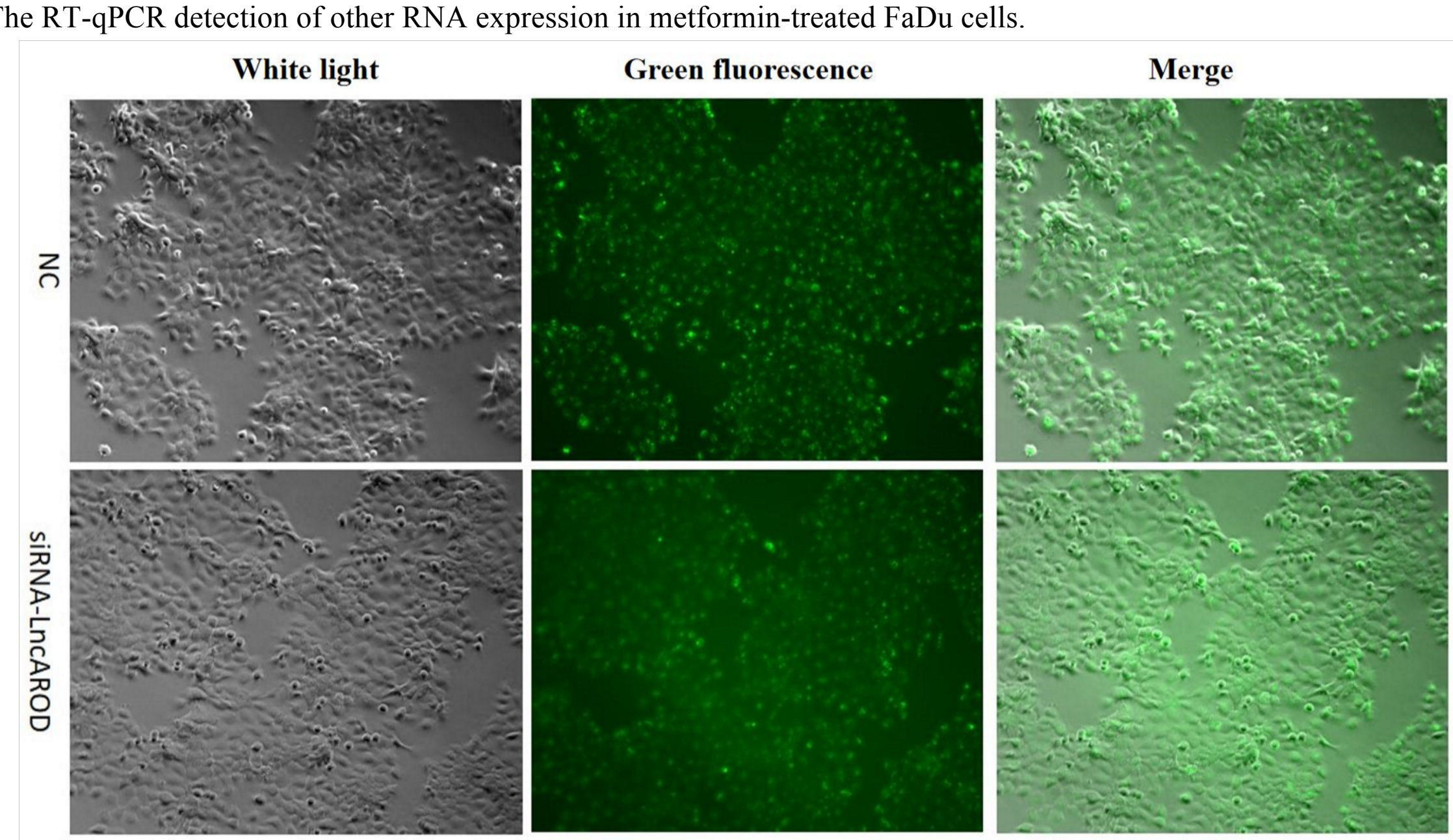

Figure S2 The green fluorescence in Fadu cells transfected was observed under fluorescence microscope

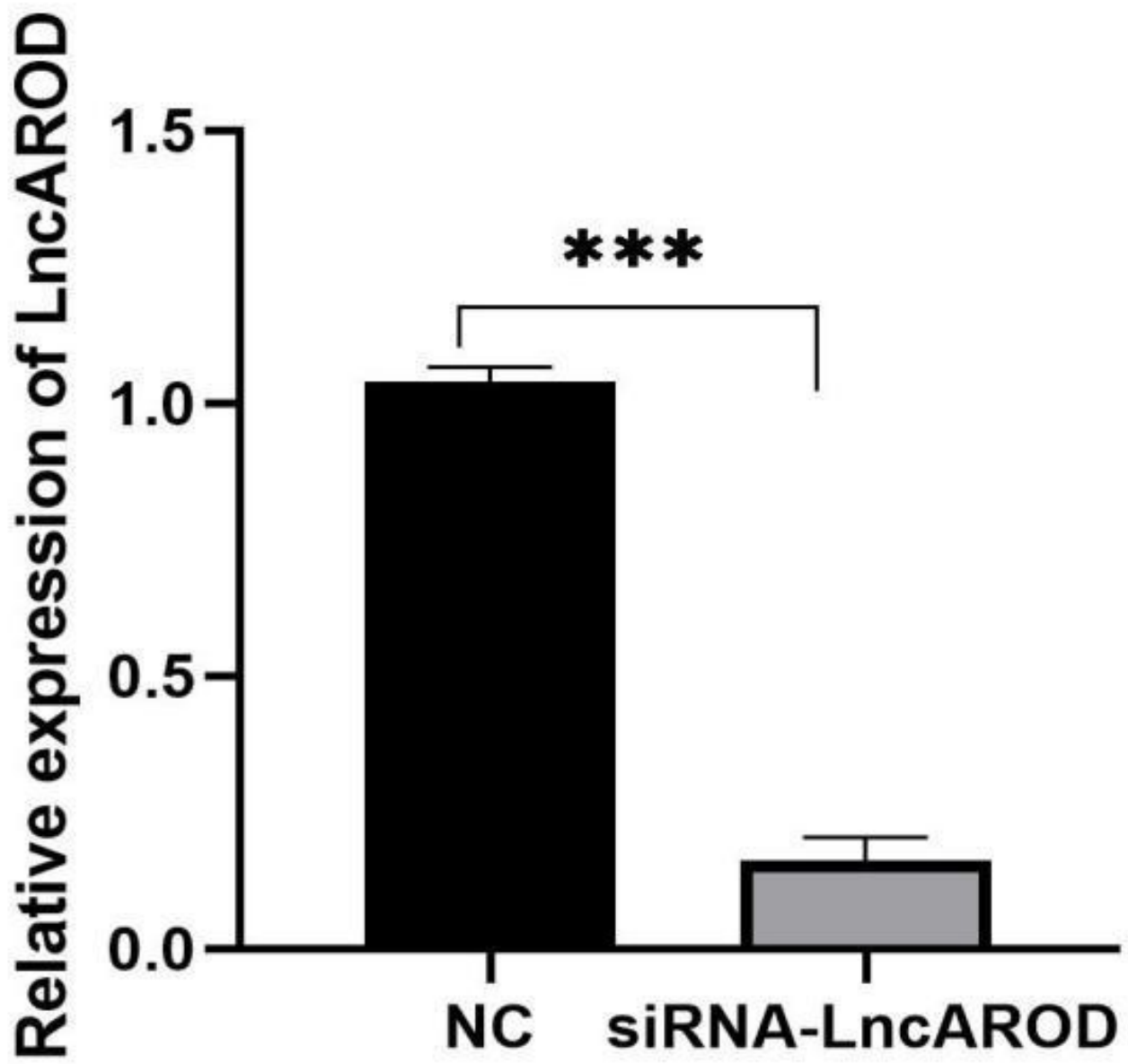

Figure S3 RT-qPCR confirmed the transfection efficiency of siRNA-LncAROD

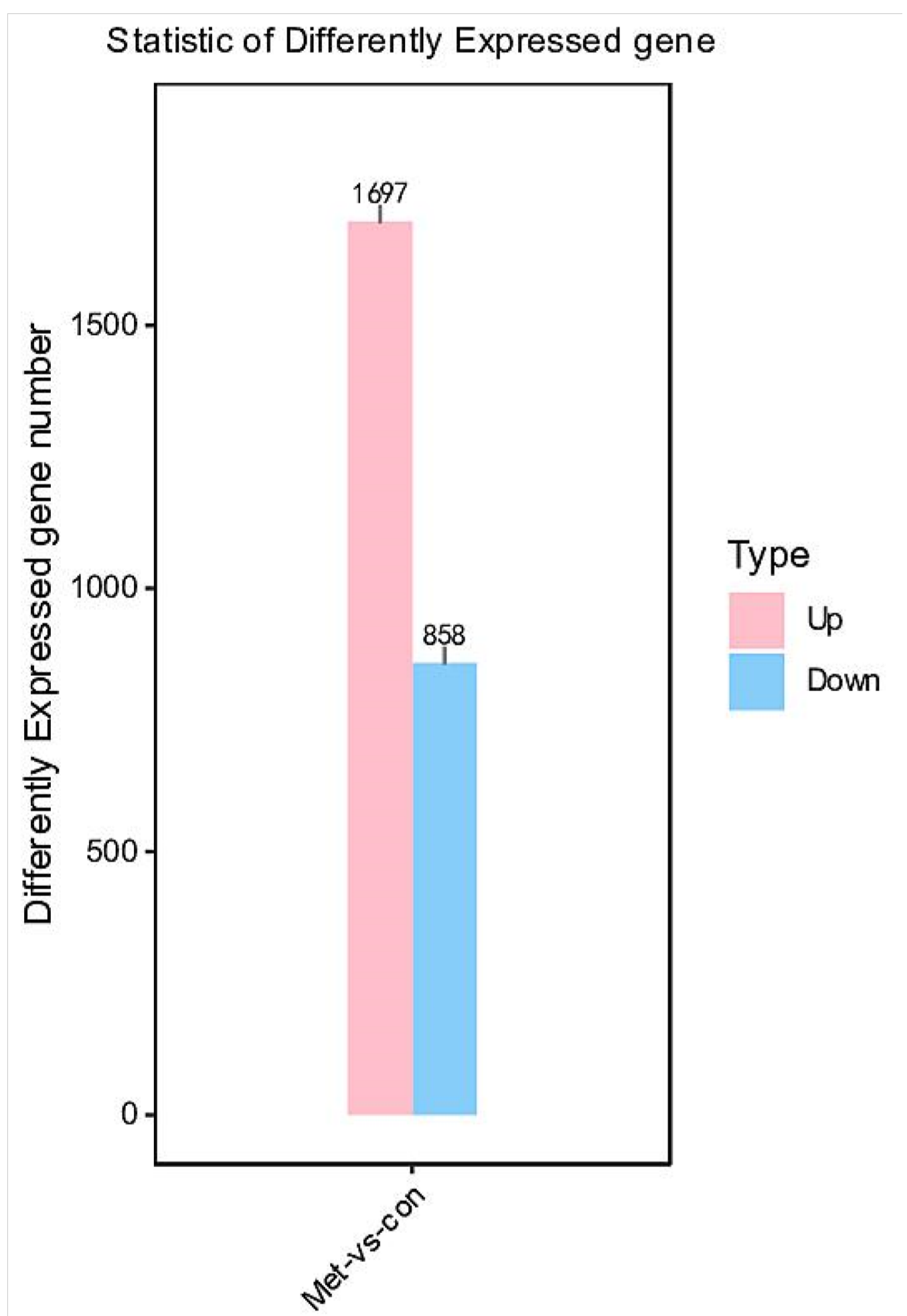

Figure S4 Bar chart of gene expression levels.
